# Natural Products Attenuating Biosynthesis, Processing, and Activity of Ras Oncoproteins: State of the Art and Future Perspectives

**DOI:** 10.3390/biom10111535

**Published:** 2020-11-10

**Authors:** Renata Tisi, Vadim Gaponenko, Marco Vanoni, Elena Sacco

**Affiliations:** 1 Department of Biotechnology and Biosciences, University of Milano—Bicocca, 20126 Milan, Italy; renata.tisi@unimib.it (R.T.); marco.vanoni@unimib.it (M.V.); 2 Bicocca Center of Science and Technology for FOOD (BEST4FOOD), 20126 Milan, Italy; 3 Department of Biochemistry and Molecular Genetics, University of Illinois at Chicago, Chicago, IL 60637, USA; vadimg@uic.edu; 4 SYSBIO-ISBE-IT-Centre for Systems Biology, 20126 Milan, Italy

**Keywords:** Ras oncogenes, Ras signaling, cancer, Ras inhibitory strategies, Ras druggable pockets, Ras inhibitors, natural products, anticarcinogenic effect

## Abstract

*RAS* genes encode signaling proteins, which, in mammalian cells, act as molecular switches regulating critical cellular processes as proliferation, growth, differentiation, survival, motility, and metabolism in response to specific stimuli. Deregulation of Ras functions has a high impact on human health: gain-of-function point mutations in *RAS* genes are found in some developmental disorders and thirty percent of all human cancers, including the deadliest. For this reason, the pathogenic Ras variants represent important clinical targets against which to develop novel, effective, and possibly selective pharmacological inhibitors. Natural products represent a virtually unlimited resource of structurally different compounds from which one could draw on for this purpose, given the improvements in isolation and screening of active molecules from complex sources. After a summary of Ras proteins molecular and regulatory features and Ras-dependent pathways relevant for drug development, we point out the most promising inhibitory approaches, the known druggable sites of wild-type and oncogenic Ras mutants, and describe the known natural compounds capable of attenuating Ras signaling. Finally, we highlight critical issues and perspectives for the future selection of potential Ras inhibitors from natural sources.

## 1. Introduction

This review focuses on the current insights and prospective developments in the therapeutic targeting of Ras oncoproteins by natural products (NPs).

Although large-volume high-throughput screening (HTS) of chemical libraries have been conducted in recent years to discover inhibitors of Ras, natural product research has not been prioritized in this field yet. Nonetheless, natural products have proven to be a valuable source of novel medicines in the last decades.

We think that the moment is appropriate for focusing on natural products in the research and development of Ras inhibitors. In fact,

(i) here is a strong need for targeted therapies to treat Ras-dependent diseases, such as most cancers, in the perspective of precision medicine. At the moment, there are few drugs, even synthetic ones, which have proved promisingly effective in clinical trials and are limited to targeting specific pathological mutants of Ras, which, although important, represent only a small percentage of those involved in human pathologies;
(ii) NPs can be successfully applied to different direct and indirect strategies of inhibition of Ras activity;
(iii) NPs represent an unlimited and still little-explored resource of chemical structures that are currently accessible thanks to the new platforms for isolation, purification, and characterization that can warrant the quality, safety, and efficacy of the active compounds.
(iv) the advancement of knowledge of the structure/function relationships of Ras proteins and their pathological variants allowed to identifiy novel druggable pockets of the proteins to be considered in the virtual/experimental screening of novel inhibitors.

We first describe the basic structure of Ras proteins, their processing, and the salient features of Ras signaling, including upstream regulators and downstream effectors, and outline the involvement of mutant Ras proteins in human diseases (Section 2, Section 3, Section 4 and Section 5). In the second part, we describe the general strategies available to inhibit Ras oncoprotein activity and signaling in cancer therapy, as well as the approaches that led to the identification of new druggable pockets in the Ras proteins (Section 6). We then describe the known NPs that inhibit Ras biosynthesis, processing and activity. On purpose, we only briefly mention compounds interfering with downstream effectors of the Ras pathway (Section 7) and outline future perspective for NPs as a valuable source for lead compounds selectively inhibiting Ras oncoproteins (Section 8).

## 2. Ras Proteins

Ras proteins are eukaryotic small guanine nucleotide-binding (G) proteins that, by cycling between the GDP-bound inactive state and the GTP-bound active state, act as molecular switches in signaling pathways regulating many cellular processes, including proliferation, growth, survival, adhesion, migration, and metabolism in mammalian cells [1].

Ras proteins are endowed with low intrinsic GTPase activity and a very slow rate of spontaneous nucleotide exchange. The Ras activation state is finely regulated, in response to different specific extracellular stimuli, by the competitive interplay of upstream regulators: Guanine nucleotide Exchange Factors (GEFs) and GTPase Activating Proteins (GAPs). GEFs activate Ras proteins by promoting nucleotide dissociation and, thereby, preferentially GDP/GTP exchange due to GTP being 10-fold more abundant in cells than GDP, while GAPs inactivate them by providing an essential catalytic group for GTP hydrolysis [2,3,4,5].

**Figure 1:**
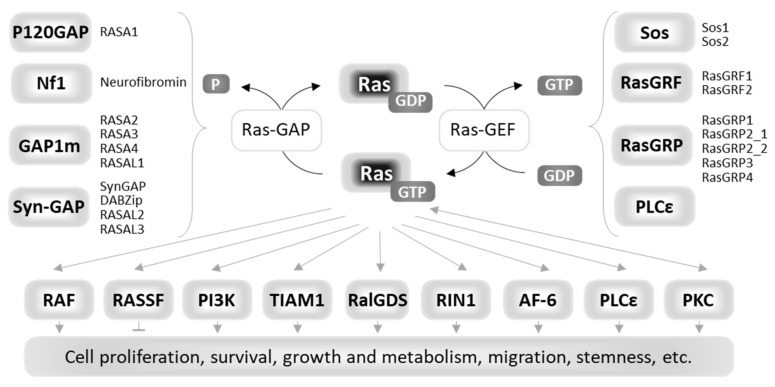
Diagrammatic representation of the functional cycle, upstream regulators, and downstream effectors of Ras proteins.

Bound nucleotides (GDP or GTP) are associated with dynamic conformational changes in the effector lobe, which contains the two regions of Ras known as switch I (residues 30–38) and switch II (residues 59–76). Together with the phosphate-binding motif (P-loop, residues 10–17), switch I constitutes the nucleotide binding pocket, while switch II is the flexible region that undergoes the main conformational changes upon nucleotide exchange [6]. In the active GTP-bound state, Ras proteins increase their affinity for many effectors that initiate downstream signal transduction [7,8]. Figure 1 shows the functional cycle of Ras proteins, their main upstream regulators (Ras GAPs and Ras GEFs), and downstream effectors, described in detail in the paragraph “Ras signaling in mammalian cells”.

## 3. Ras Processing and Subcellular Localization

In human cells, three RAS genes (HRAS, NRAS, and KRAS), encode four homologous but functionally distinct isoforms, HRas, NRas, KRas4A, and KRas4B, the two latter ones deriving from alterative splicing of the KRAS gene [9,10].

The isoforms share 90% of sequence identity in the G-domain (residues 1–166), which is composed of an effector lobe (residues 1–86) and an allosteric lobe (87–166). As mentioned above, the effector lobe contains the nucleotide-binding and the effector-interacting sites, while the allosteric lobe plays an intramolecular communication role by connecting the active site of the effector lobe to membrane-interacting residues [11].

Ras isoforms mainly differ in the carboxyl-terminal hypervariable region (HVR) that contains sites for posttranslational modifications (PTM). This region is responsible for membrane tethering of Ras proteins that is required for correct membrane trafficking and localization and the function of each isoform [12,13].

While the HVRs of all Ras isoforms are C-terminally prenylated (farnesylated or geranylgeranylated) and methylated, only the HVRs of HRas, NRas, and KRas4A bear additional palmitoyl moieties [14]. The HVR of KRas4B is not palmitoylated but, instead, contains a unique poly-lysine patch that assists with membrane association. These C-terminal modifications anchor Ras in the membrane bilayer and promote the formation of dimers at the plasma membrane and signaling [15,16]. Ras dimers and higher-order assemblies act as platforms for organization of multiprotein signaling complexes [17,18,19,20,21,22]. Importantly, isoform-specific HVRs direct Ras proteins to different microdomains of the plasma membrane or endomembranes (endoplasmic reticulum, Golgi, endosomes, and mitochondria) [23,24], where diverse signaling cascades are initiated.

The HVR of KRas4B can be phosphorylated on serine residues by protein kinase C (PKC) and protein kinase A (PKA) [25]. Phosphorylation of KRas4B by PKC affects the signaling and trafficking of KRas4B to internal membranes that include the endoplasmic reticulum and the outer mitochondrial membrane [26]. Moreover, S181-phosphorylated KRas4B is capable of Bcl-xL-dependent binding to inositol trisphosphate receptors and blocking IP_3_ receptor-mediated calcium release [27]. In addition, HVR ubiquitination on K170 in KRas4B leads to its dissociation from the plasma membrane and reduction in MAPK signaling [28,29]. Thus, the unique amino acid sequences, together with Ras isoform-specific post-translational modifications at the hypervariable C-terminus, ensure the intricate regulation of signaling outputs in response to extracellular stimuli. This is an emerging area of research that is poised for rapid expansion in the near future [30].

## 4. Ras Signaling in Mammalian Cells

In mammals, Ras proteins are major hubs at the crossroads of many signaling pathways, which allow cells to respond to different stimuli by the means of the activation/inactivation of several tissue- and context-specific intracellular processes. This versatility depends on a network of multiple upstream regulators and downstream effectors of Ras proteins subjected to complex spatiotemporal organization (Figure 1).

### 4.1. Upstream Regulators of Ras

Ras activity is regulated by many accessory proteins, with Ras-specific GEF or GAP activity, each responsive to a specific stimulus, such as a ligand-activated surface receptor or an oscillation in the intracellular levels of a specific cellular component (i.e., calcium, phosphoinositides, etc.). These upstream regulators are multidomain proteins in which homologous but distinct GEF or GAP domains are flanked by other functional regions with specific catalytic or docking activity [5]. The presence of multiple domains in GEF and GAP proteins allows not only to regulate Ras activity in response to specific stimuli but, also, to coordinate Ras signaling with other signaling pathways and cellular events via complex regulatory mechanisms also involving competing intermolecular and intramolecular interdomain interactions.

In mammalian cells, there are three main Ras GEF families (Sos, Ras GRF, and Ras GRP) sharing a catalytic CDC25^Sc^ homologous domain [31,32].

Sos proteins are ubiquitously expressed and transmit the extensively studied mitogenic signal downstream of the cell surface tyrosine kinase growth factor receptors, including the epidermal growth factor receptor (EGFR) and insulin receptor [33]. Activation of the Ras-specific GEF activity of Sos is subjected to complex regulatory mechanisms that require its growth factor-dependent recruitment to the plasma membrane via Grb2 and changes in the PA and PIP_2_ levels [31,34,35], intramolecular rearrangements in order to release the Ras GEF domain from inhibitory constraints exerted by its flanking regions [36,37,38,39], and the allosteric interaction of a Ras GTP with a distal site within the catalytic domain [40,41].

Ras GRF proteins are mainly expressed in the central nervous system and are responsive to glutamate receptors; G-protein-coupled receptors; or changes in intracellular calcium concentration, Trk, and cannabinoid receptors [42]. Finally, Ras GRP proteins are expressed in blood cells and are responsive to nonreceptor tyrosine kinases [43]. Additionally, the phospholipase PLCε contains a CDC25Sc homologous domain and promotes Ras activation downstream various receptor tyrosine kinase and G-protein-coupled receptor agonists by distinct pathways [44,45].

The human genome encodes 14 Ras GAP proteins grouped in five families (RASA1/P120GAP, Nf1, GAP1IP4BP, SynGAP, and IQGAP) with little sequence similarity outside the catalytic region [45,46]. Notably, IQGAP members have a not catalytically competent Ras GAP domain due to amino acid substitutions. In contrast to Ras GEFs, there is considerably less information regarding the GTPase activating proteins that negatively regulate Ras activity. Structural studies based on P120GAP [3,4] elucidate the catalytic mechanism promoting the hydrolysis of Ras-bound GTP, but the role of the other domains of Ras GAP proteins and the signaling cascades controlling their recruitment and activation have not yet been fully clarified. The best-characterized functional Ras GAP is neurofibromin, whose loss of function mutants play an established role in the autosomal dominantly inherited complex disease, known as neurofibromatosis type I, which predisposes to tumors along the nervous system [47].

### 4.2. Downstream Effectors of Ras

The best-characterized Ras effectors are RAF, PI3K, and RalGDS. RAF are multidomain serine/threonine kinases (ARAF, BRAF and RAF1, AKA CRAF) that mediate MEK-ERK signaling (also known as the MAP kinase cascade), controlling, through phosphorylation, the activation of a plethora of targets [48], mainly transcriptional factors (e.g., Myc) that drive the transcriptional rewiring of many cellular processes, including cell migration, proliferation, differentiation, and survival. [49,50]. Upon stimulation by growth factors, active RAS recruits RAFs to the plasma membrane via their Ras-binding domains (RBDs) and promotes the formation of functionally asymmetric RAF homo- and heterodimers in which one monomer-usually, BRAF-allosterically stimulates the kinase activity of the other.

Phosphatidylinositide-3-kinase α (PI3Kα) is primarily a lipid kinase that phosphorylates phosphatidylinositol (4,5)-*bis*-phosphate (PIP_2_) to phosphatidylinositol (3,4,5)-*tris*-phosphate (PIP_3_) and activates Akt/mTOR signaling, promoting cell growth and metabolism rearrangement, cell survival, and the prevention of apoptosis and autophagy [51]. In particular, Ras stabilizes the catalytic subunit of PI3K (p100), facilitating PIP_2_ binding at the active state. Afterwards, RTKs recruit PI3K to the plasma membrane and induce conformational changes in the regulatory subunit (p85). The mechanistic aspects of Ras-mediated activation of Raf and PI3K are discussed in detail elsewhere [52].

RalGDSs are nucleotide exchange factors specific for RalA and RalB small GTPases (about 50% identity with Ras), which can interact with activated Ras GTP through their Ras association (RA) domains, also known as Ras-interacting domain (RID). They link Ras to the activation of Ral GTPases, which regulate endocytosis, exocytosis, actin cytoskeletal organization, cell migration, and gene expression, by interacting with multiple functionally distinct downstream effectors, as reviewed elsewhere [53,54,55].

Different isoforms of PKC differently affect Ras signaling [56,57,58], contributing to Ras downstream or upstream events. Wang and colleagues demonstrated that PKC can phosphorylate Ser181 in the C-terminal HVR region of the KRas isoform (not in other isoforms), preventing its binding with Ca-calmodulin, thereby affecting the noncanonical Wnt pathway through FZD receptors [59] and its multiple functions, as in cytoskeletal dynamics, establishment of planar cell polarity (PCP), and stemness [60,61].

Besides the above-described Ras effectors, there are several others, such as RIN [62], TIAM [63], PLCε [64], RASSF [65,66,67,68], AF6 [69,70], and IMP [71], which impressively complicate the signaling network downstream of Ras, even including complex negative feedbacks. For example, members of the RASSF family promote Ras-induced apoptosis and senescence and oppose Ras-induced mitogenic and survival signaling [72]. It should also be noted that other proteins are involved in Ras signaling regulation, such as UBIAD1, which interacts with the C-terminal region of HRas and modulates its trafficking from the Golgi apparatus to the plasma membrane [73] or the prenyl-binding protein PDEδ, regulating the correct localization and signaling by farnesylated KRas [74]. The crosstalk and relative balance between all these pathways determine the cellular responses, which require a system level approach to be understood. Notably, the activation of specific effector pathways in a cell containing multiple Ras effectors depends on the balance between Ras affinity for each effector and the effector concentration in a specific subcellular domain at the plasma membrane or endomembranes [24,75,76].

## 5. Ras Mutants in Human Diseases

Due to the critical role of Ras signaling in the regulation of cell proliferation, growth, differentiation, survival/apoptosis, metabolism (energy and redox homeostasis), adhesion, migration, and stemness, the deregulation of Ras activity has a driving role in the pathogenesis of several human diseases, including developmental disorders, known as RASopathies [77,78], and several types of cancer.

RASopathies are a group of phenotypically overlapping syndromes (Costello syndrome, Noonan syndrome, LEOPARD syndrome, and cardio-facio-cutaneous (CFC) syndrome) causing facial abnormalities, impaired growth and development, heart defects, mental retardation, and, in some instances, a predisposition to specific cancers, due to germline mutations in the components of the Sos-Ras-MAPK axis [77,78].

Mutations in RAS genes can also be detected in nearly one-third of human tumors, including the deadliest ones. KRAS is the most frequently mutated isoform (21%), followed by mutations in NRAS (8%) and in HRAS (3%) (www.sanger.ac.uk/genetics/CGP/cosmic/). A mutationally activated KRas oncoprotein is present in almost all pancreatic ductal adenocarcinomas and in up to 50% of colorectal cancers. The large majority of gain-of-function missense mutations that constitutively activate the Ras oncoproteins map at codons 12 (89%), 13 (9%), and 61 (1%) [79], which are the key participants in the interplay between Ras, nucleotides, and modulators (see also below). Other noncanonical codons mutated in cancer at a low frequency are 19, 117, and 146 [80]. Each oncogenic mutation alters the functional cycle of Ras through a distinct mechanism depending on the conformational change induced by the presence of the mutated amino acid. For example, the G12V substitution abolishes the intrinsic and GAP-mediated GTP hydrolysis due to interference with the allosteric switch [81,82], while G13D mutation determines the self-sufficiency in nucleotide dissociation, even maintaining the sensitivity to GEFs and at least one GAP [81,82,83,84,85,86]. The Q61L mutation reduces the intrinsic, in both free and Raf-bound Ras, and GAP-mediated GTP hydrolysis and accelerates the nucleotide exchange [82]. Regardless of the activation mechanism, all oncogenic Ras mutants show an altered residence time in the GTP-bound active state [81] and aberrantly transduce downstream signals contributing to tumor onset, maintenance, and progression [87], impinging on most cancer hallmarks [88], such as growth signal-independent sustained proliferation, resistance to apoptosis, the ability to migrate and to invade/metastasize, the ability to promote angiogenesis, and the ability to elude the immune response, as previously reviewed [89]. Oncogenic KRAS activation also induces significant changes in cell metabolism, including enhancement in glucose transport and aerobic glycolysis [90,91,92] that determine the acquisition of the hyperglycolytic phenotype known as the Warburg effect [93,94], anaplerotic usage of glutamine [95,96,97], altered sulfur amino acid metabolism [98], altered mitochondrial morphology and function, and the production of large amounts of reactive oxygen species (ROS) [99,100]. Ras GAPs and members of the RASSF family constitute a barrier to Ras-dependent transformation in cells. However most Ras oncoproteins are insensitive to GAP, and loss-of-function of Ras GAPs or RASSFs is common in tumors [72].

## 6. Strategies for Inhibiting Ras Oncoproteins Biosynthesis, Processing, Activity, and Signaling in Cancer Therapy

**Figure 2:**
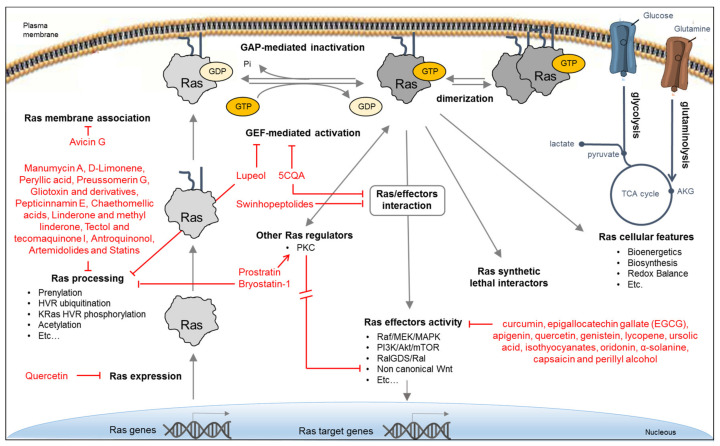
Approaches for inhibiting Ras oncoproteins biosynthesis, processing, activity, and signaling. Indirect Ras inhibitory approaches include the interference with different processes: expression of Ras oncogenes, Ras processing and membrane localization, activity of Ras regulators, activity of downstream effectors, Ras-dependent cellular features, and the activity of synthetic lethal interactors. Direct Ras inhibitory approaches include the interference with Ras/GEF interaction and exchange activity, Ras/effector interaction, and Ras dimerization.

Due to the critical role of Ras oncoproteins in cancer, many efforts, mostly promoted by the RAS initiative (https://www.cancer.gov/research/key-initiatives/ras), have been devoted to explore different direct and indirect strategies for attenuating their aberrant signaling, as recapitulated in several recent reviews [101,102,103] and schematically depicted in Figure 2. Bioactive natural products identified in some of these strategies are reported in the figure.

### 6.1. Indirect Strategies

The high nucleotide affinity and the apparent lack of pockets on the Ras surface capable of accommodating potential drug candidates lead to the exploration of indirect strategies to inhibit Ras oncoproteins functions in cancer cells. These strategies include the targeting of Ras regulators, such as exchange factors [104,105,106,107,108] or PKC [26,59], and the inhibition of Ras effectors directly involved in tumor maintenance and progression, such as Raf, MEK, and PI3K [109,110,111,112].

Alternative approaches target features induced by oncogenic Ras signaling and not shown by normal cells, i.e., metabolic and redox alterations. Indeed, even though the metabolic changes induced by RAS oncogenes sustain the enhanced cell growth of Ras cancer cells, paradoxically, some of these cause addiction to specific nutrients (i.e., glutamine) or to specific metabolic activities and become the potential Achilles’ heel of cancer cells, which can be exploited in targeted therapies [113]. Similarly, the increased levels of ROS that, on one hand, promote proliferation and survival signaling, induce enhanced oxidative stress sensitivity in Ras-transformed cells [98].

Another approach to selectively kill cancer cells expressing RAS oncogenes is to target their synthetic lethal interactors [114,115,116,117], namely the molecular elements whose function is essential only in a Ras-transformed context [115].

In addition, the inhibition of Ras oncogene expression, also exploiting recent advances in gene therapy methodologies [118,119], and methods aimed at impairing the membrane localization of Ras proteins required for their function were widely explored [13,120,121,122,123,124]. These last ones include the inhibition of Ras HVRs-processing involved in association with the membrane.

### 6.2. Direct Strategies: Ras Proteins as Pharmacological Targets

Unfortunately, attempts at all strategies indirectly targeting Ras have disappointing clinical activity against Ras-driven cancers and have not yet yielded any approved drugs. Therefore, the necessity to identify novel tools for Ras drugging is compelling.

Recently developed tools for highly processive and high-throughput virtual and experimental screening allow analyzing libraries of thousands of compounds on different targets quickly and efficiently.

Whenever an experimental high-throughput screening (HTS) system is available for evaluating the affinity or the potency of a library of compounds towards a clinical target, neither virtual screening nor a priori knowledge of the 3D structure of the natural compound may be necessary. This approach was used to identify irreversible inhibitors of Ras^G12C^ oncogenic mutant [125] (see below) or small molecules that bind to the Ras/Sos complex and perturb Ras signaling [126] or Sos inhibitors that block RAS activation [104]. The experimental approach, coupled with comparative structural investigation of the inhibitor-bound and inhibitor-free proteins, reveals that Ras can adapt to the interactors, showing novel, interesting druggable pockets that challenge the previously proposed notion of Ras as an “undruggable” target [127]. Several compounds were identified that either can interfere with Ras/GEF binding or with nucleotide exchange activity [83,106,128,129,130,131,132,133,134,135,136] or can inhibit Ras effectors binding [137,138] or both [139]. A recent approach tackles the ability of Ras proteins to dimerize [21], thus blocking Raf activation.

#### 6.2.1. Molecular Issues in Targeting Ras

As we described above, Ras proteins are molecular switches with overlapping interaction surfaces with their upstream and downstream partners, along their signaling cascade. In order to design/identify selective and specific inhibitors of these proteins, dynamic structure-activity relationships have to be carefully considered. In particular, the P-loop and the extremely flexible switch I and switch II regions constitute the site of binding for nucleotides, regulators, and effector proteins. Oncogenic mutations indeed also appear in this region, but they do not cause any dramatic overall structural changes, partly due to the intrinsic molecular flexibility of these segments [140]. Decoding conformational heterogeneity is the first mandatory step in any drug design attempt towards such flexible targets.

**Figure 3:**
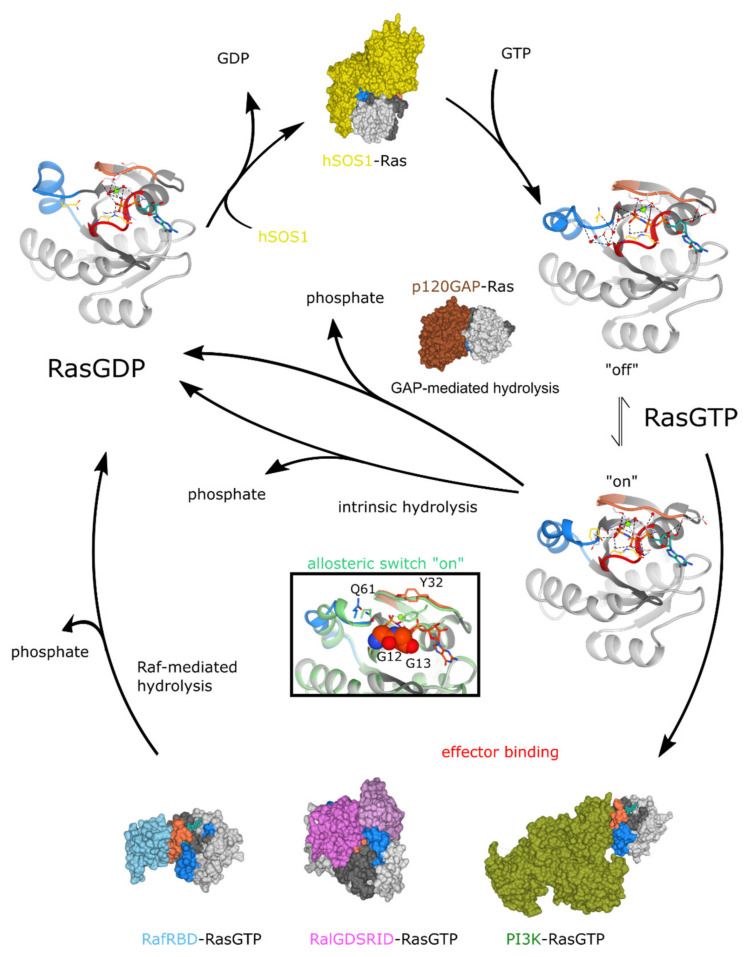
Ras proteins cycle. Ras proteins (gray) nucleotide exchange is catalyzed by Ras Guanine nucleotide Exchange Factors (GEFs) such as hSOS1 (in yellow). Ras GTP can assume different conformations due to an allosteric switch that is in the dynamic equilibrium between an “off” state (PDB ID: 5p21) and an “on” state (PDB ID: 3K8Y), both spontaneously and in dependence of Ras GTPase Activating Proteins (GAPs) (such as p120GAP in brown and PDB ID: 1WQ1) or Ras effectors binding. A detail of the “on” conformation (in green) is shown in the insert, superimposed on the “off” conformation: the rearrangement in the position of residues Q61 in switch II and Y32 in switch I is evident. The presence of the two glycines in positions 12 and 13 (represented as spheres) are mandatory to allow this rearrangement, which is fundamental for the catalytical mechanism of GTP hydrolysis. Three of the main effectors of Ras are shown in complex with Ras GTP: Raf Ras-binding domain (RBD) is in light blue (PDB ID: 4G0N), RalGDS Ras-interacting domain (RID) are in pink and purple (PDB ID: 1LFD), and PI3K catalytic subunit is in green (PDB ID: 1HE8). Note that RalGDS binds Ras GTP as a heterotetramer. All of the effectors bind Ras only in the active form due to their higher affinity for the Ras GTP conformation of the effector lobe (dark grey). Ras switch I is in orange, and switch II is in blue. The nucleotide is in green sticks, while Q61 and Y32 are shown as yellow sticks in Ras proteins structures.

At the molecular level, the presence of GTP rather than GDP allows the formation of a network of hydrogen bonds among the γ-phosphate and switch I, P-loop, and switch II residues (see Figure 3), together with several water molecules. This network drives both switches into a more ordered conformation that allows binding to Ras GAPs and Ras effectors. Wittinghofer and coworkers described that the presence of the glycines at positions 12 and 13 is mandatory in order to allow the insertion of the catalytic “arginine finger” provided by the GAP into the optimal position for hydrolysis catalysis [3]. The conserved Ras Gln61, which is located in the switch II region, is likely involved in the activation of the water molecule for an attack of the γ-phosphate of GTP. The GAP is also responsible for the correct positioning of Gln61 for catalysis, conferring an allosteric effect. These molecular details reveal why the hotspots for Ras oncoproteins mutations reside in these particular residues.

The binding domains for the Raf1 effector partially overlaps with that for GAP through switch I but not switch II (Figure 3), yet its affinity to Ras is much higher than the Ras affinity for p120GAP [141]. Ras/Raf interaction is controlled by intrinsic hydrolysis, where switch I is modulated by the binding of Raf, and switch II is positioned for catalysis by the allosteric switch [141] (Figure 3). In this case, the switch I residue Tyr32 is situated in a position similar to that of the GAP arginine finger. Gln61 is positioned by the allosteric switch in a critical position for stabilizing the transition state of the reaction catalyzed by Ras in the absence of GAP when the allosteric switch is in the “on” state (Figure 3, insert).

#### 6.2.2. Druggable Pockets in Ras Proteins

**Figure 4:**
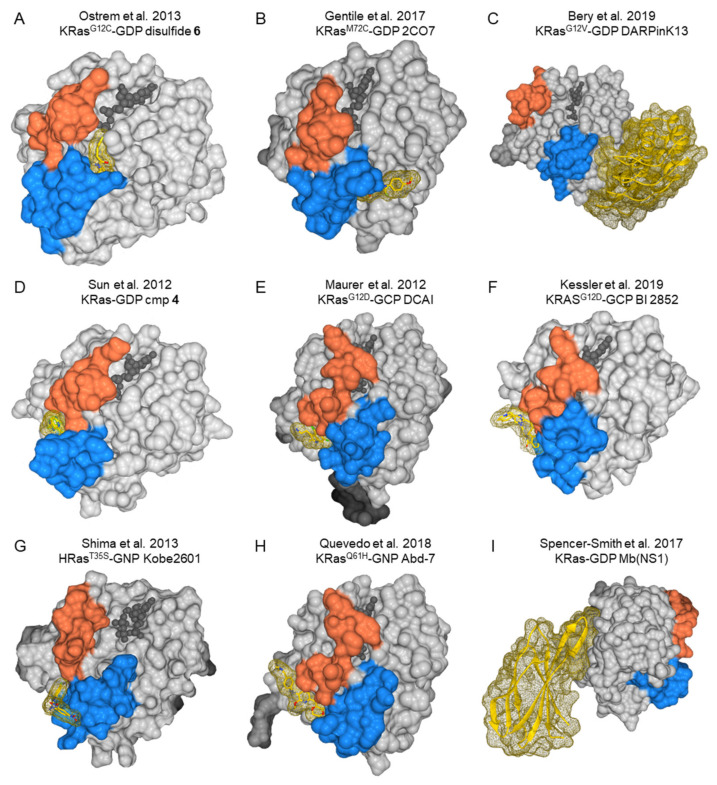
Structures of Ras proteins binding to different inhibitors. Switch I is in orange, and switch II in blue. Guanine nucleotide is in dark grey balls. Inhibitors are represented as yellow sticks and their surface as a yellow mesh. (**A**) KRas^G12C^ GDP bound to disulfide **6** (PDB ID:4luc) [125], (**B**) KRas^M72C^ GDP bound to 2CO7 (PDB ID:5vbm) [149], (**C**) KRas^G12V^ GDP bound to DARPinK13 (PDB ID:6h46) [151], (**D**) KRas GDP bound to cmp 4 (PDB ID:4epv) [131], (**E**) KRas^G12D^ GCP bound to DCAI (PDB ID:4dst) (Maurer et al., 2012) [131], (**F**) KRAS^G12D^ GCP bound to BI 2852 (PDB ID:6gj8) [139], (**G**) HRas^T35S^ GNP bound to Kobe2601 (PDB ID:2lwi) [137], (**H**) KRas^Q61H^ GNP bound to Abd-7 (PDB ID:6fa4) [138], and (**I**) KRas GDP Mb(NS1) (PDB ID:5e95) [152].

The pioneering studies of Ostrem and colleagues in 2013 contributed to overturn the undruggable view of Ras proteins demonstrating the presence of an allosteric pocket in a common oncogenic mutant KRas^G12C^ [125]. Crystallographic studies revealed the formation of a new pocket that is not apparent in previous structures of Ras beneath the flexible effector binding switch II region upon the irreversible binding of small molecules (Figure 4A). Their inability to bind the GTP state of KRas^G12C^ is compensated by the quite unaffected intrinsic GTPase activity of this oncogenic variant, allowing a slow transition of the GTP- to GDP-bound form [81,142]. The optimized versions of these inhibitors, selectively directed against KRas^G12C^, are the first and only drugs so far directly targeting Ras in clinical trials, with very promising antitumor effects in KRAS^G12C^-positive lung and colon adenocarcinoma patients [143,144,145]. The selectivity of these compounds can hardly be extended to the other oncogenic variants, since it exploits the peculiar reactivity of the Cys12 residue. For this reason, the need to obtain effective inhibitors for the other Ras oncoproteins remain an extremely topical in cancer therapy.

Although multiple Ras GDP crystal structures reveal that the Switch II residues are more mobile in the GDP state than in the GTP state, NMR studies suggest that Ras GTP can adopt multiple conformational states to accommodate effector binding and GTPase activities [146,147,148]. A fragment-based tethering screen with an engineered cysteine mutant of KRas^M72C^ was performed in order to discover new scaffolds that could adapt to switch II pocket in both nucleotide states. This screen yielded the fragment 2C07 [149] (Figure 4B), which expands the switch II pocket inhibition to both nucleotide states by stabilizing Ras GDP and preventing PI3K activation by Ras GTP. Notably, a similar pocket was proposed to be targeted by the natural compound 5CQA, which is actually able to interfere with HRas GTP binding to Raf1RBD [150].

A phage display selection of a diverse designed ankyrin repeat proteins (DARPins) library, followed by immunoassays with KRAS^G12V^ to isolate hits, allowed to identify macromolecules that specifically inhibit the KRAS isoform by binding to an allosteric site encompassing the region around KRAS-specific residue histidine 95 at the helix α3/loop 7/helix α4 interface [151] (Figure 4C).

Fragment-based lead discovery (FBLD) has exploited NMR and surface plasmon resonance for the detection of ligand-protein interactions, even at millimolar affinities. Characterization of the site through a combination of structural studies and biophysical and biochemical examinations allowed the identification of previously undetected pockets on Ras protein effector lobes. Sun and colleagues [131] identified low-affinity KRas^G12D^ GDP-binding fragments by a NMR-based screening. These molecules also bind wild-type K- and HRas at a hydrophobic pocket, located between the α2 helix of switch II (60–74) and the central β sheet of the protein (Figure 4D), that is occupied by Tyr-71 in the apo-Ras crystal structure. The presence of these molecules interferes with Ras/GEF binding and, thus, inhibits the Sos-catalyzed nucleotide exchange. Through an NMR-based fragment screen, Maurer and colleagues [130] delineated a similar pocket (Figure 4E), confirming that compound-binding interferes with the Ras/SOS interactions.

Another alternative approach is based on the knowledge-based rational analysis. Structure-based drug design (SBDD) was applied to discover BI2852 [139] (Figure 4F), a KRas inhibitor that binds to a pocket between switch I and II with nanomolar affinity. BI2852 binds to a pocket present in both the active and inactive forms of KRas, blocking all GEF, GAP, and effector interactions and leading to inhibition of downstream signaling and proliferation in KRas mutant cells.

Another attempt to target Ras GTP took advantage of the determination of a more druggable Ras GTP alternative conformation [146,148] in dynamic equilibrium with the previously known conformation. This approach led to the successful discovery of a novel class of small-molecule compounds able to sequester Ras GTP from its multiple effector molecules and, moreover, display antitumor activity on a xenograft of human colon carcinoma cells carrying the G12V-mutated *KRAS* gene [137] (Figure 4G).

A different approach to targeting the same protein-protein interaction (PPI) interface was undertaken by Quevedo and colleagues [138] (Figure 4H) using an intracellular anti-mutant Ras antibody fragment as a competitor in a small-molecule library screen for identifying Ras-binding compounds. Again, the structure-based design allowed to optimize the initial hits, resulting in potent Ras-binding compounds that prevent Ras-effector interactions and inhibit endogenous Ras-dependent signaling.

A previously unrecognized functionally critical region of Ras was identified in the α4-β6-α5 region (Figure 4I) outside the effector lobe, which can be targeted by a synthetic binding protein (monobody) termed NS1 that binds with high affinity to both GTP- and GDP-bound states of H- and KRas [21], thus specifically inhibiting oncogenic Ras-mediated signaling and transformation. NS1 binding to Ras disrupts Ras dimerization/nanoclustering, which, in turn, blocks CRAF:BRAF heterodimerization and activation.

## 7. Natural Products Targeting Biosynthesis, Processing, and Activity of Ras Oncoproteins

Natural products that have been identified as indirect Ras inhibitors with different mechanisms of action are described in Section 7.1 and listed in Table 1 (NPs targeting Ras expression and regulation) and Table 2 (NPs targeting Ras processing), while Section 7.2 contains a brief description of NPs inhibiting Ras effectors.

Natural products directly targeting Ras are described in Section 7.3 and listed in Table 3.

### 7.1. NPs Indirectly Targeting Ras Function

#### 7.1.1. NP Inhibiting Ras Expression

• Quercetin

Quercetin is a dietary flavonoid found in tea, onions, grapes, wines, and apples, and the anticancer activities of this compound have been previously explored in breast and colon cancer cells [153]. Quercetin reduced the expression of numerous prostate tumor-associated microRNAs (miRNAs) [154]. Quercetin regulated the cisplatin sensitivity of human osteosarcoma cells by modulating the miR-217-*KRAS* axis [155]. Consistently, quercetin reduced the steady-state levels of K-, H-, and NRas mRNAs and proteins in both colon cancer cell lines and primary colorectal tumors [156].

#### 7.1.2. NPs Inhibiting Ras Regulation and Membrane Association

• Avicin G

A more indirect effect is obtained with Avicin G, a family of natural plant-derived triterpenoid saponins from *Acacia victoriae*, which mislocalizes KRas from the plasma membrane and disrupts the plasma membrane spatial organization of KRas and HRas oncoproteins by depleting phosphatidylserine and cholesterol contents, respectively, at the inner plasma membrane leaflet [157]. Avicin G also inhibits oncogenic K- and HRas signal outputs and the growth of KRas-addicted pancreatic and non-small cell lung cancer cells. Avicin G also perturbs lysosomal activity and disrupts cellular localization and the activity of sphingomyelinases, resulting in altered cellular sphingomyelin levels and distribution.

• Bryostatin-1

Bryostatin-1 is a cyclic macrolide isolated from the marine bryozoan *Bugula neritina* that acts as a protein kinase C (PKC) agonist, activating PKC isozymes at nanomolar concentrations [158,159].

PKC-mediated phosphorylation of the C-terminal segment of KRas4B regulates its association with the plasma membrane. In particular, bryostatin-1 induces a rapid translocation of KRas to intracellular membranes such as the endoplasmic reticulum (ER) and Golgi apparatus but, also, to the outer mitochondrial membrane where KRas stimulated Bcl-Xl-dependent apoptosis [26]. Bryostatin-1 is in clinical development as an antileukemic agent and is also in phase II clinical trials against melanomas, lymphomas, and renal cancer [160].

• Prostratin

Prostratin is a phorbol ester found in the bark of the mamala tree of Samoa, *Homalanthus nutans* (Euphorbiaceae), acting as an activator of atypical PKCs. It can efficiently reduce the interaction of KRas and CaM, rewire Wnt/Ca^2+^ signaling, and suppress malignancy mediated by oncogenic KRas in pancreatic cancers [59].

#### 7.1.3. NPs Targeting Ras Processing

As described, Ras proteins must be isoprenylated at a conserved cysteine residue in order to properly exert their biological function. An intermediate in mevalonate pathway, most likely farnesyl pyrophosphate, is the donor of this isoprenyl group. Since mevalonate is the precursor of various products essential to mammalian cells, such as dolichols, ubiquinones, heme A, and cholesterol, the strategy of using inhibitors of the mevalonate pathway to block the transforming properties of *RAS* oncogene proved to be difficult. Specific farnesyl transferase (FTase) inhibitors were developed, but this strategy collided with the activity of geranylgeranyl transferase (GGTase), allowing an alternative way for Ras targeting to the membranes [13]. Several natural products interfering either with the mevalonate pathway or with farnesyl transferase activity itself were characterized (for reviews, see [161,162]). Here, we summarize some compounds among the more efficient against cancer cell proliferation recently characterized (Table 2). Some of them led to anticancer compounds that are in clinical trials, such as antroquinolol [163].

• Manumycin A

Manumycin A is a natural macrolide antibiotic isolated from *Streptomyces parvulus* and acts as a potent peptidomimetic inhibitor of Ras farnesylation [164,165,166,167]. Manumycin A significantly inhibits the proliferation and migration of vascular smooth muscle cells (VSMCs), reduces the amount of Ras protein localized at the cytoplasmic membrane, inhibits the phosphorylation of MAPK, and disorganizes the actin fibers [168]. In addition, manumycin A decreases exosome biogenesis in prostate cancer cells and in myofibroblasts primarily via the targeted inhibition of Ras/Raf/ERK1/2 signaling [169,170].

• D-Limonene and peryllic acid

D-Limonene is a common monoterpene found in essential oils of orange, lemon, mandarin, lime, grapefruit, and many other plants, with antiproliferative, apoptosis-inducing, and chemo-preventive effects and, as similar monoterpenes, inhibits Ras prenylation [171,181,182]. The related compound peryllic acid is able to inhibit both FTase and GGTase [183].

• Preussomerin G

The preussomerins and deoxypreussomerins are phenolic fungal metabolites extracted from the coprophilous fungus *Preussia isomera* and the endophytic fungus *Harmonema dematioides* with FTase and GGTase inhibitory properties [172,184,185,186]. Low-toxicity synthetic esters derived from these compounds required reductive activation, specifically at the cancer cells, resulting from hypoxia and the overexpression of reductases. The anticancer activity was determined in cancer cell lines with reported reductase activity, such as BC-1 cells and NCI-H187 [187].

• Gliotoxin and derivatives

Gliotoxin is a sulfur-containing mycotoxin, produced by various pathogenic fungi, including Aspergillus fumigatus, that inhibit Ras farnesylation and cell growth [163,164]. Some derivatives were developed as GGTase-specific inhibitors [188].

• Pepticinnamin E

The natural product pepticinnamin E was reported to inhibit protein farnesyl transferases and cell proliferation almost 30 years ago [173,189]. Pepticinnamin E contains a rare N-terminal cinnamoyl moiety, as well as several nonproteinogenic amino acids, which mimic the two substrates of FTase, CAAX, and FPP. Its biosynthetic pathway has only recently been characterized due to the loss of the original producer organism [190]. A library of 51 analogs was generated from pepticinnamin E and screened for FTase inhibitory activity [191].

• Chaethomellic acids

Chaethomellic acids are a class of alkyl dicarboxylic acids, isolated from *Chaetomella acutiseta*. They are potent and highly specific farnesyl-pyrophosphate (FPP) mimic inhibitors of Ras FTase with lower specificity for GGTases [174,192]. Long-term treatment with chaethomellic acid A can attenuate the Ras-dependent progression of renal fibrosis in a murine model of chronic kidney diseases [175].

• Linderone and methyl linderone

The cyclopentenediones linderone and methyl linderone isolated from the fruits of *Lindera erythrocarpa* (Lauraceae) showed FTase inhibitory and antitumor activity [176,193].

• Tectol and tecomaquinone I

Tectol and the related compound tecomaquinone I were isolated in a screening for FTase inhibitors; tectol also exhibited significant activity against the human leukemia cell lines HL60 and CEM [177,194].

• Antroquinonol

A compound with anti-inflammatory activities extracted from the mycelium of *Antrodia camphorate* antroquinonol has been shown to exert anticancer effects in lung cancer, liver cancer, and leukemia by inhibiting the activity of both Ras FTase and GGTase [163,178,195].

• Arteminolides

Arteminolides (A-D) are dimeric sesquiterpene lactones isolated from *Artemisia* spp. with an inhibitory activity on FTase [196,197]. These compounds and other similar sesquiterpene lactones from *Artemisia* inhibited tumor cell growth in a dose-dependent manner [198,199]. In particular, arteminolide C blocked the in vivo growth of human colon and lung tumor xenografts [179].

• Statins

Several statins, comprising natural ones (lovastatin and simvastatin), efficiently inhibited KRas protein trafficking from the cytoplasm to the cell membrane of pancreatic cancer cells due to depletion of the mevalonate pathway intermediates [180].

### 7.2. NPs Inhibiting Ras Effectors

• Several Compounds

Many Ras effectors play a relevant role in the onset and progression of Ras-dependent disorders and, therefore, represent attractive therapeutic targets for drug development. Several inhibitors of Ras-ERK signaling have been developed, including Raf inhibitors and MEK inhibitors, as reviewed [200]. Several natural products were reported to inhibit ERK signaling, although the mechanisms of action are often unclear. Among these, we can enlist sulforaphane, epigallocatechin gallate (EGCG), isothiocyanates, genistein, and perillyl alcohol (see [201] for a review). Additionally, the inhibition of the PI3K-AKT-mTOR pathway has been widely experimented, even with natural products such as lycopene, curcumin, resveratrol, genistein, apigenin, oridonin, α-solanine, and capsaicin [202,203,204,205,206].

### 7.3. NPs Targeting Ras Activity Directly

• 5-O-caffeoylquinic acid (5-CQA)

The first natural compound reported to directly target Ras activity was a chlorogenic acid and was identified on the basis of its structural resemblance to previously identified synthetic Ras inhibitors [128,136]. The chlorogenic acids (CGAs) occur ubiquitously in food, representing the most abundant polyphenols in the human diet. Particularly high levels of chlorogenic acid (5-O-caffeoylquinic acid, 5-CQA) were found in coffee beans used to prepare green coffee and, after roasting, black coffee, a widespread drink worldwide. A number of CGA beneficial biological effects, including anti-inflammatory activity, anticarcinogenic activity, and protection against neurodegenerative diseases, were reported. Its mechanism of action is based on the inhibition, upon direct binding to the target, of Ras interaction with both activators and effectors. In addition, viability and MAPKs activation/phosphorylation assays performed on KRas^G13D^ expressing breast cancer cells, MDA-MB-231, suggested its capability of reducing cancer cell growth [150].

• Lupeol

The triterpenoid lupeol was reported to inhibit farnesyl transferase [209] and, thus, to inhibit the growth of KRas mutant cancer cell lines but not of wild-type KRas-expressing cells [207]. Lupeol was identified as a KRas directly binding compound in an in silico screening of a library of triterpenoid class of molecules and its binding results in inhibition of the GDP/GTP exchange [207].

• Swinhopeptolides

Two new cyclic depsipeptides named swinhopeptolides A and B were isolated from the marine sponge *Theonella swinhoei* cf. *verrucosa*, collected from Papua, New Guinea. These compounds contain 11 diverse amino acids and 13 carbon polyketide moieties attached at the N-terminus. They can impede the interaction between Ras and Raf, a serine/threonine protein kinase. Swinhopeptolides A and B showed significant inhibition of the Ras/Raf signaling pathway, with half maximal inhibitory concentration (IC_50_ values) in the micromolar range [208].

## 8. Conclusion and Perspectives: Natural Products as a Source of Selective Inhibitors of a Ras Oncoproteins

Although various strategies to inhibit Ras have been explored over three decades, success has only recently been achieved in human clinical trials-in particular, with small molecules capable of directly inhibiting the activity of the Ras^G12C^ oncogenic mutant. However, effective inhibitors are yet to be found for the other oncogenic variants.

**Figure 5:**
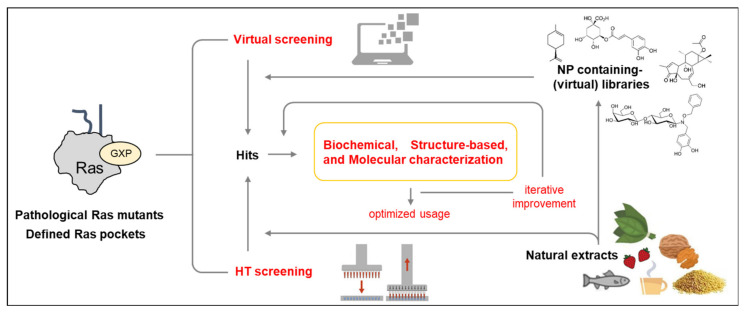
Approaches for the identification and development of Ras inhibitors from natural sources. HT: high-throughput.

Since natural products provide a virtually limitless source of structurally novel, highly diverse natural compounds, they would be a promising approach to discover novel molecules with a higher affinity for oncogenic Ras proteins. Three points are worth mentioning on this subject:

(i) the improvement of techniques that allow to isolate, purify, and structurally characterize new molecules of natural origin, often already available in large libraries [210,211];
(ii) the simultaneous development of experimental and computational approaches for their high-throughput screening (HTS) on targets of clinical relevance;
(iii) the availability of virtual screening allowing to identify the structurally most promising compounds for a target of interest, thereby reducing the research costs.
(iv) Both structure-based virtual screenings and HTS approaches with Ras oncoproteins as targets will now be able to take advantage of the newly discovered druggable pockets available in specific oncogenic Ras isoforms and mutant proteins to isolate, characterize, and iteratively improve Ras-specific inhibitors (Figure 5).

Such knowledge can be put at work to tailor the design of the screen and to characterize hits obtained from libraries or natural extracts, as well as to define optimal Ras isoforms or mutant targets for the NPs. By way of example, a combination of NMR spectroscopy, molecular docking, surface plasmon resonance, and assays on a Ras-dependent cancer cell line allowed the identification and characterization of 5-CQA [150], while in silico screening of a triterpenoid library identified lupeol as a KRas-binding compound that can be considered as a lead compound for the further development of Ras inhibitors [207]. The structural and functional traits of each pathogenic Ras variant (and binding pockets) can make them more or less prone to binding specific pharmacophores or drugs and can be exploited to identify molecular determinants conferring specificity for the oncogenic variant, which could become interesting candidates for drug leads. Some of the binding pockets, such as the K-RAS-specific allosteric site bound by the DARPins [151], may be difficult to target with small molecules (Figure 4C). Others, such as the region outside the effector lobe targeted by the NS1 monobody (Figure 4I), could be an interesting target for the screening of natural compound binders, since small compounds can target this region [21].

We hope that this review will encourage researchers working on NPs to join efforts with researchers in the Ras field, allowing to identify, characterize, and develop a new set of natural products (and, possibly, second-generation derivatives of the same) effectively downregulating the biosynthesis, processing, and activity of Ras oncoproteins.

## Figures and Tables

**Table 1:** List of natural compounds indirectly affecting Ras oncoproteins activity.

Compound	Target	Mechanism of Action	Source	Models	Ref.
Quercetin 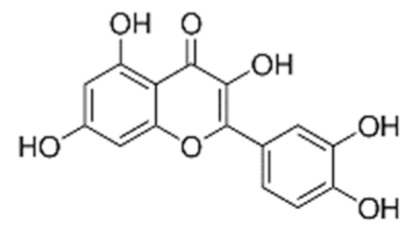	K-, N-, and HRAS	indirect inhibition of expression	red grapes and red wine	colon cancer cells	Zhang et al., 2015 [155]
Prostratin 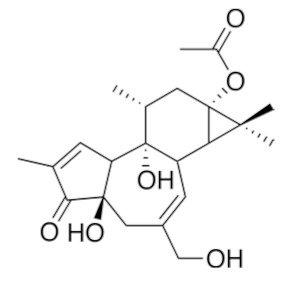	KRas	Inhibition of CaM interaction	mamala tree of Samoa, *Homalanthus nutans*	pancreatic cancer murine models	Wang et al., 2015 [59]
Avicin G	K- and HRas	indirect delocalization	*Acacia victoriae*	cells expressing mGFP-KRas^G12V^	Garrido et al., 2020 [157]
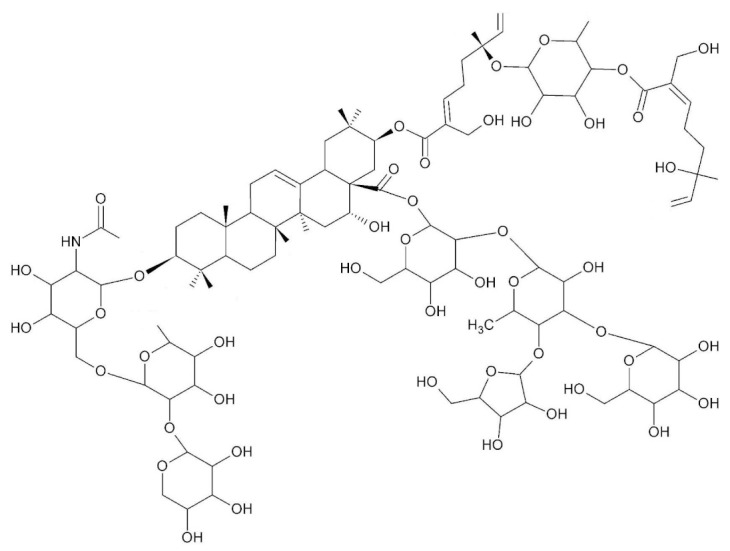					
Bryostatin-1 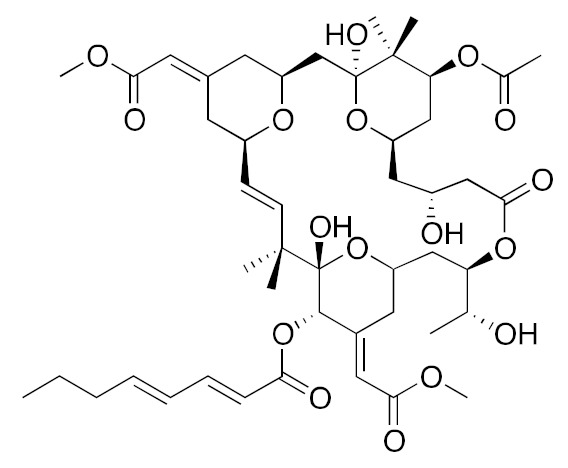	K-Ras4B	direct delocalization	marine organism *Bugula neritina*		Bivona et al., 2006 [26]

**Table 2:** List of natural compounds indirectly affecting Ras oncoprotein activity by interfering with Ras prenylation.

Compound	Source	Models	Ref.
Manumycin A	*Streptomyces parvulus*	castration-resistant prostate cancer (CRPC) C4-2B	Datta et al., 2017 [169]
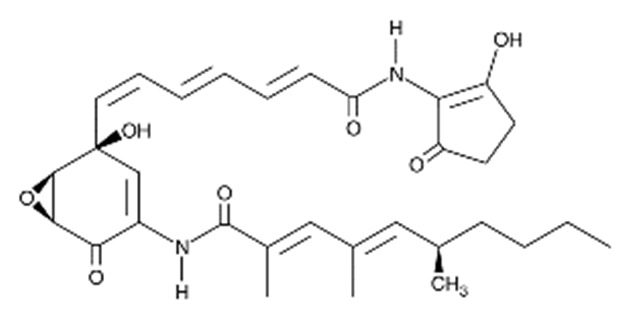			
D-Limonene 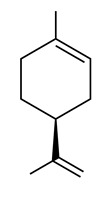	orange peels; other plants essential oils	W 1-38. CACW, A549 and PaCa cells	Chen et al., 1998 [171]
Preussomerin G 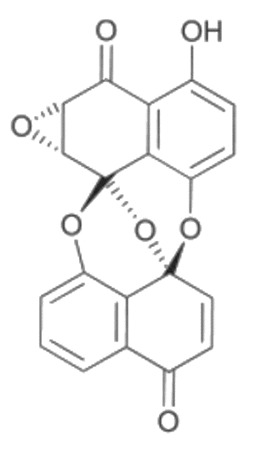	*Preussia isomera* and *Harmonema dematioides*	BC-1 and NCI-H187 cells	Singh et al., 1994 [172]
Gliotoxin 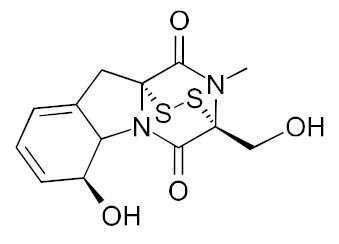	*Aspergillus*, *Trichoderma*, and *Penicillium*	human colon carcinoma (LoVo) cells	Nagase et al., 1997 and Saha et al., 2009 [164,165]
Pepticinnamin E 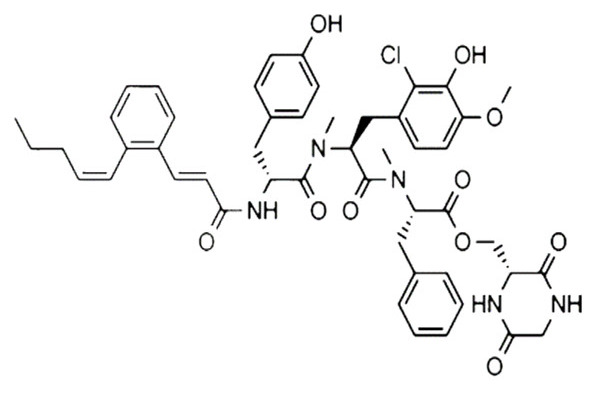	*Actinobacteria bacterium*	Kidney Vero cells	Omura et al., 1993 [173]
Chaetomellic acid A 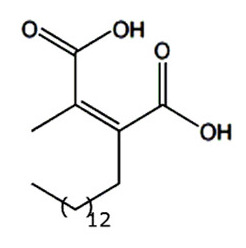	*Chaetomella acutiseta*	murine model of renal fibrosis	Gibbs et al. 1993; Nogueira et al. 2017 [174,175]
Methyl linderone 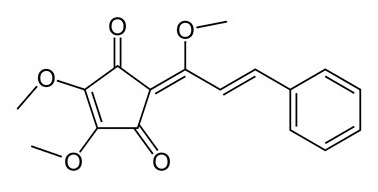	fruits of *Lindera erythrocarpa*	human breast cancer cells MCF-7	Yoon et al., 2020 [176]
Tectol 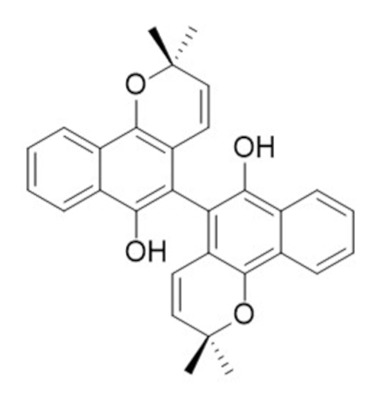	Brazilian *Lippia sidoides*	HL60 (human promyelocytic leukemia) and CEM (human acute lymphoblastic leukemia)	Costa et al., 2001 [177]
Antroquinonol 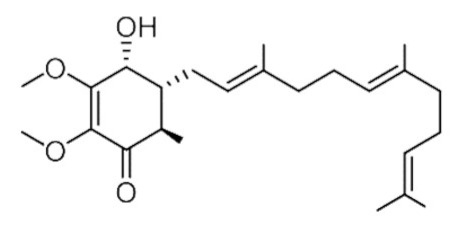	*Antrodia camphorata*	Human lung cancer (A549 and H838), liver cancer (HepG2 and Hep3B), and leukemia (K562 and THP-1) cells	Ho et al., 2014 [178]
Artemidolide C 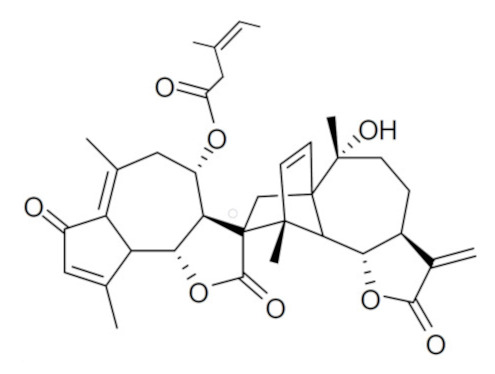	*Artemisia* spp.	SW620 (colon), MDA-MB-231 (breast), HCT116 (colon), and MCF7 (breast)	Lee et al., 2003 [179]
Statins (lovastatin, simvastatin) 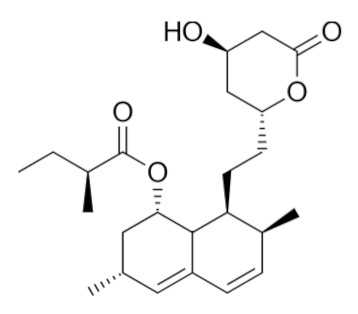	*Aspergillus terreus*	pancreatic cancer cells	Gbelcová et al., 2017 [180]

**Table 3:** List of natural compounds directly affecting Ras oncoproteins activity.

Compound	Target	Mechanism of Action	Source	Models	Ref.
5CQA, 5-O-caffeoylquinic acid 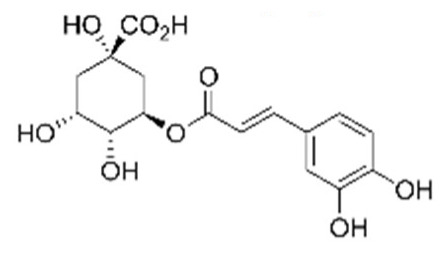	HRas	direct inhibition of nucleotide exchange and Raf1 binding	coffee	KRas^G13D^ breast cancer cells	Palmioli et al., 2017 [150]
Lupeol 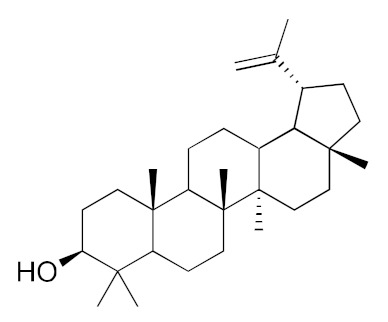	KRas	direct inhibition of nucleotide exchange	many edible fruits and vegetables	human and murine KRAS-driven cancer models	Ganaie et al., 2020 [207]
Swinhopeptolides 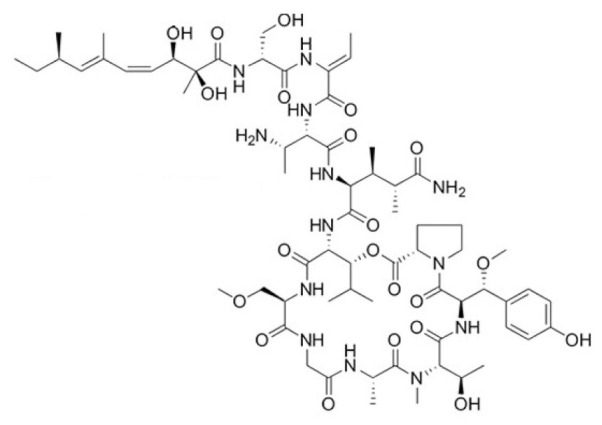	unknown	direct inhibition of Raf1 binding	Papua New Guinea marine sponge *Theonella swinhoei* cf. *verrucosa*	unknown	Kim et al., 2020 [208]
